# Supplementary material for: Content-rich biological network constructed by mining PubMed abstracts
Source: BMC Bioinformatics. 2004 Oct 8;5:147. doi: 10.1186/1471-2105-5-147 (PMC528731; doi:10.1186/1471-2105-5-147)
Supplement: Additional File 2 — The original results of the above study (non-essential files are deleted to keep the file size under the limit set by BMC bioinformatics). [file 1471-2105-5-147-S2.bz2 › chilibotAdditionalFile2/dip05/723ID9180258E18063/html/SECG_SECY.html]

 


 **SECG** and **SECY** 
  
Found 41 abstracts in PubMed, retrieved 05.  
 

 What does Google say? 
 PDF only 
| .edu only 

---

**Interactive relationship** (e.g. stimulation, inhibition, etc)

**Inhibitory relationship**- In addition, mutations in  **secG**  were isolated as suppressors against a cold sensitive mutation secY104 affecting the TM4 C3 boundary of  **SecY** .  Ref: 12809499 Biochemistry, 2003
**Neutral relationship**- These results collectively suggest that a C2 TM3 P2 TM4 C3 region of  **SecY**  serves as an interface with  **SecG** .  Ref: 12809499 Biochemistry, 2003
- Thus, the C2 C3 region of  **SecY**  is in the proximity of the N terminal half of the  **SecG**  cytoplasmic loop.  Ref: 12809499 Biochemistry, 2003
- These  **SecY**  Cys residues also formed a disulfide bond with an engineered cytosolic cysteine at position 28 of  **SecG** .  Ref: 12809499 Biochemistry, 2003

**Non-interactive relationship** (e.g. studied together, co-existance, homology, etc.)

- Integral membrane components  **SecY** , SecE, and  **SecG**  of protein translocase form a complex in the Escherichia coli plasma membrane.  Ref: 12809499 Biochemistry, 2003
- These prokaryotic eukaryotic proteins include 1  **SecY**  Sec61alpha, 2 SecE Sec61gamma, 3  **SecG**  Sec61beta, 4 Ffh SRP54 and 5 FtsY SRP receptor subunit alpha.  Ref: 12507766 Biochim Biophys Acta, 2003
- Experiments using spheroplasts revealed the physical proximity of P2  **SecY**  and the C terminal periplasmic region of  **SecG** .  Ref: 12809499 Biochemistry, 2003
- SecYEG, a complex of integral membrane proteins  **SecY** , SecE, and  **SecG**  in the cytoplasmic membrane, constitutes a pathway for polypeptide movement.  Ref: 12812064 Yi Chuan Xue Bao, 2003
- **SecY** , SecE and  **SecG**  form a heterotrimer, which functions as a protein translocation channel in Escherichia coli.  Ref: 12655407 Mol Genet Genomics, 2003
- Experiments using inverted membrane vesicles revealed specific cross linkings between a cysteine residue placed in the C2 or C3 domain of  **SecY**  and the cytosolic lysine Lys26 near the first transmembrane segment of  **SecG** .  Ref: 12809499 Biochemistry, 2003
- Identification of a  **SecY**   **SecG**  Interface.  Ref: 12809499 Biochemistry, 2003
- **SecY**   **SecY**  and  **SecY**   **SecG**  contacts revealed by site specific crosslinking.  Ref: 12220653 FEBS Lett, 2002
